# Defining the marker and developmental trajectory of myeloid-derived suppressor cells in aging by single-cell transcriptomics

**DOI:** 10.1038/s41514-025-00317-x

**Published:** 2025-12-24

**Authors:** Yaru Su, Ruimin Wu, Haochen Ai, Zhaoming Zhong, Lin Zou, Zihan Wang, Kewu Tu, Lingzheng Tang, Jiawen Gao, Yusheng Huang, Congrui Liao, Guanhai Zeng, Hongyang Zhang, Jian Jin, Siyuan Zhu

**Affiliations:** 1 Department of Ophthalmology, Nanfang Hospital, Southern Medical University, Guangzhou, China; 2 The First School of Clinical Medicine, Southern Medical University, Guangzhou, China; 3 Division of Spine Surgery, Department of Orthopedics, Nanfang Hospital, Southern Medical University, Guangzhou, China; 4 Department of Orthopedics, Taihe People’s Hospital, Guangzhou, China

**Keywords:** Cancer, Cell biology, Computational biology and bioinformatics, Immunology

## Abstract

Myeloid-derived suppressor cells (MDSCs) are recognized as a key mediator of immunosuppression in aging, which induce immunosenescence and increase elderly people’s susceptibility to infections, cancers, autoimmune diseases, and degenerative diseases. However, the commonly used MDSC markers overlap with those defining healthy and normal neutrophils or monocytes, which makes it challenging to distinguish MDSCs from their myeloid counterparts, and hampers deeper understanding of the pathophysiological functions of MDSCs. In this study, we compared MDSCs from aged mice to young controls using single-cell RNA sequencing. We established MDSC-specific gene signature, which revealed the general characteristics of MDSCs during aging, and thus facilitating distinguishing them from normal myeloid cells. Experimental study revealed that CD300c may serve as a specific marker for improved detection and enrichment of MDSCs in aging. CD11b^+^Gr1^+^CD300c^+^ cells demonstrated a robust ability of T cell suppression. The universality and applicability of MDSC-specific gene signature have also been demonstrated in human myeloid cells. We also found that MDSCs from aged individuals shared the similar developmental trajectory with their myeloid counterparts, and may develop from mature myeloid cells, both in mice and human beings, which has been reported by a limited number of studies. Overall, our work extends the understanding of MDSCs in aging process.

## Introduction

It is now well known that the aging process profoundly remodel the immune system, reducing the activity of both innate and adaptive immunity, resulting in the increased risk of cancers, autoimmune diseases, infections in the old populations^1^. A chronic low-grade inflammation is one of the important hallmarks of the aging process, which is termed as inflammageing^2^. Inflammageing is closely related to age-related immune deficiency, generally known as immunosenescence^2,3^. Accumulated researches have reported that immunosenescence might be caused by an increased activity of immunosuppressive cells rather than cellular senescence^4^.

Myeloid-derived suppressor cells (MDSCs) are described as a loosely defined group of heterogeneous neutrophil- and monocyte-like myeloid cells, which originate from hematopoietic stem cells (HSCs) through the myelopoietic pathway^5^ and are increasingly recognized as one of the key mediators of immunosuppression in various conditions^6,7^. Two major subsets were determined based on their phenotypic and morphological features: granulocytic or polymorphonuclear MDSCs (PMN-MDSCs) and monocytic MDSCs (M-MDSCs)^6^. Previous studies showed that inflammageing induces myelopoiesis in the bone marrow, which not only increases generation of mature myeloid cells, such as monocytes, macrophages, and neutrophils, but also immature myeloid progenitors and MDSCs^8^. It is now known that aging process is associated with a significant increase in the presence of MDSCs in the bone marrow, blood, spleen, and peripheral lymph nodes^8–11^. The expanded MDSCs drive immunosuppression and induce immunosenescence, recent studies have shown that MDSCs from aged individuals could inhibit antigen-induced T cell proliferation, impair natural killer (NK) cell and dendritic cell (DC) function and suppress B cell response^7^. In addition, MDSCs could also induce the proliferation and differentiation of regulatory T (Tregs) and B (Bregs) cells, thereby establishing an intact immunosuppressive network^4,12^. MDSCs could mediate immunosuppression by stimulating the expression of arginase 1 (ARG1), arginase 2 (ARG2) and indoleamine 2,3-dioxygenase (IDO), which serve to deplete essential amino acids vital for T cell proliferation, thereby exerting a potent inhibitory effect on the immune response^13^. In addition, MDSCs also secrete reactive oxide species (ROS) and nitric oxide (NO), alongside a spectrum of immunosuppressive cytokines such as interleukin-10 (IL-10) and transforming growth factor-β (TGF-β)^7,14^. Collectively, these mechanisms perform pivotal functions in the immunosuppression capabilities of MDSCs.

It is now known that human PMN-MDSCs have been described as CD11b^+^CD15^+^HLA-DR^low^CD66b^+^, M-MDSCs as CD11b^+^CD14^+^CD33^+^HLA-DR^low/neg^^7^. Their mouse counterparts have been defined as CD11b^+^Ly6G^+^ and CD11b^+^Ly6C^+^ Ly6G^low^, respectively^7^. However, these markers are also expressed by the classical or mature monocytes and neutrophils, this will increase the difficulty to distinguish MDSCs from normal myeloid cells, which limits our perceptions of MDSCs^15^. In recent years, with the development of higher-dimensional cytometric studies and single-cell RNA sequencing (scRNAseq) technology, researchers have described the extensive heterogeneity and the markers of MDSCs in different pathological conditions^16–18^. Nevertheless, the unique molecular features of MDSCs in aging process are now unclear, and it remains elusive whether MDSCs represent a unique subpopulation of myeloid cells that differ from their young, healthy counterparts.

In the present study we conducted scRNAseq analysis of myeloid cells collected from old and young mice. We described the development trajectory, specific surface marker and unique gene signature of MDSCs during aging and validated the findings in human single-cell datasets simultaneously, thus facilitating distinguishing them from normal myeloid cells.

## Results

### Identifying MDSCs from neutrophilic and monocytic lineages by single-cell transcriptome analysis

**Fig. 1:**
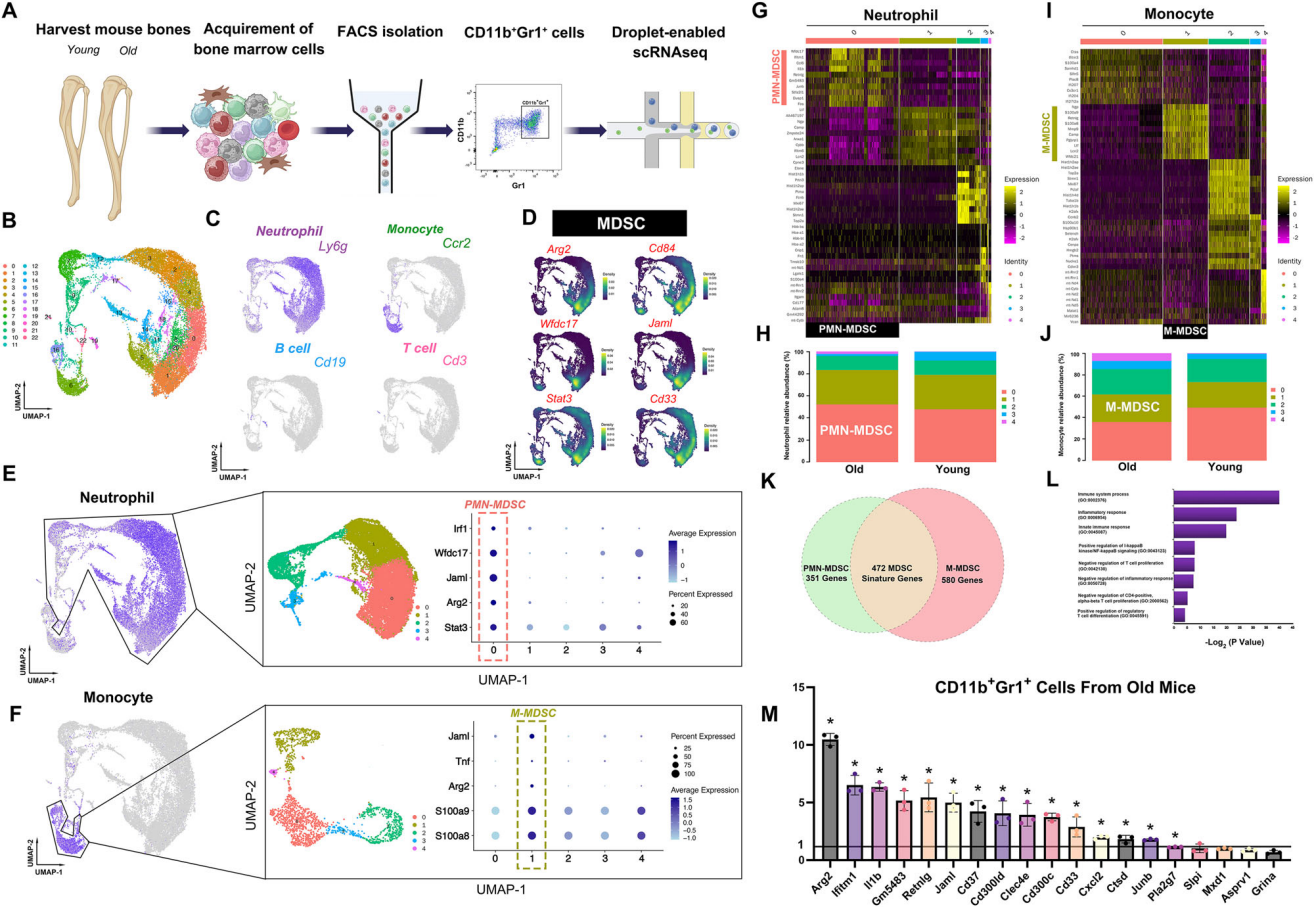
Single-cell transcriptome analysis revealed MDSC signature genes. **A** Experimental design schematic. In summary, CD11b^+^ Gr1^+^ myeloid cells were sorted from the bone marrow of both young and aged mice by FACS. And then all samples were processed using the 10X Genomics Chromium platform. **B**, **C** A total of 23,143 cells were finally analyzed. The UMAP projection result showed 23 distinct clusters in CD11b^+^ Gr1^+^ myeloid cells, and the main cell types included neutrophils, monocytes, T cells and B cells, based on the expression of marker genes. **D** The widely acknowledged MDSC marker genes Arg2, Cd84, Wfdc17, Jaml, Stat3 and Cd33 were utilized to identify both PMN-MDSCs and M-MDSCs, derived respectively from neutrophils and monocytes. **E** PMN-MDSCs were identified using the MDSC marker genes Arg2, Stat3, Jaml, Wfdc17, Irf1 in neutrophils. **F** M-MDSCs were identified using the MDSC marker genes S100a8, S100a9, Arg2, Tnf, Jaml in monocytes. **G** Heat map showing top 10 marker genes for each neutrophil subset. **H** The percentage of PMN-MDSCs found in the neutrophils of aged mice was higher than that of young mice. **I** Heat map showing top 10 marker genes for each monocyte subset. **J** The percentage of M-MDSCs found in the monocytes of aged mice was higher than that of young mice. **K** The Venn diagram illustrated the quantity of genes that were significantly differentially expressed in PMN-MDSCs and M-MDSCs, showing an overlap of 472 genes shared between these two MDSC subsets. **L** Gene ontology analysis of the MDSC signature genes. **M** RT-qPCR validated the expression levels of some MDSC signature genes. Data were presented as the mean ± SD. *P < 0.05.

To distinguish MDSCs from normal neutrophils or monocytes, scRNAseq was employed to analyze and compare the gene expression patterns of bone marrow-derived myeloid cells isolated from aged and young mice. In summary, bone marrow cells (excluding red blood cells) were extracted from the tibias and femurs of both aged and young mice. Subsequently, these cells were sorted using fluorescence-activated cell sorting (FACS) to isolate the CD11b^+^Gr1^+^ myeloid lineages and then profiled by the scalable droplet-mediated scRNAseq system (Fig. 1A). The scRNAseq libraries were generated using the 10X Genomics Chromium platform. After stringent filtering and quality control (Figure S1), a total of 23,143 cells were sequenced, including 14,281 cells from aged mice and 8862 cells from young mice. According to recently reported studies and previously established marker genes^16^, we identified the main cell types (Fig. S2) and determined the marker genes of each cluster (Supplementary Data 1). As shown in Fig. 1B-C, neutrophils constituted a predominant proportion (Cluster 0–5, Cluster 7–15, Cluster17, Cluster 20, Cluster 22), while monocytes occupied the second-largest share (Cluster 6 and 16). In addition, a small number of T cells (Cluster 21) and B cells (Cluster 19) were also identified. It was reported that Arg2, Il1b, Cd84, Wfdc17, Jaml, Stat3 and Cd33, among others, were immunosuppressive factors or markers of MDSCs^7,13,16,19^, in the present study, we found that Clusters 0, 1, 5, and portions of Cluster 6 exhibited heightened expression levels of the MDSCs-related genes (Fig. 1D). Since the expressions of the MDSCs-related genes in neutrophils and monocytes displayed a dispersed or non-specific pattern, we extracted the neutrophilic or monocytic lineage respectively, and reconducted the clustering analysis (Fig. 1E, F, Fig. S3, Fig. S4). This led to the identification of a distinct PMN-MDSC cluster (Cluster 0 within the neutrophils, Fig. S3A, B) and a distinct M-MDSC cluster (Cluster 1 within the monocytes, Figure S4A, B), both of which exhibited relatively high expression levels of the MDSCs-related genes (Fig. 1E, F). From the steps above, we have preliminarily identified PMN-MDSCs and M-MDSCs as distinct clusters that are distinguishable from their normal myeloid counterparts, and this will help to describe the molecular signature of MDSCs and find the specific marker in the following study.

### Identification of signature genes of MDSCs that differ from normal myeloid lineages

Having successfully segregated PMN-MDSCs from normal neutrophils and M-MDSCs from normal monocytes, we subsequently conducted differential expression analysis in Seurat to elucidate the distinct transcriptional profiles of PMN- and M-MDSCs. This analysis will enable us to identify the signature genes of MDSCs derived from the bone marrow of aged mice.

In the present study, 823 marker genes specific to PMN-MDSCs and 1052 marker genes specific to M-MDSCs were identified (Supplementary Data 2, Supplementary Data 3), which suggested that MDSCs differ substantially from their normal myeloid counterparts (Fig. 1G, I). Additionally, we observed elevated proportions of PMN-MDSCs and M-MDSCs in the bone marrow neutrophils and monocytes of aged mice, respectively, when compared to their young counterparts (Fig. 1H, J). The identified PMN- and M-MDSC marker genes were then intersected to obtain MDSC signature genes, and finally 472 shared genes were obtained (Fig. 1K, Supplementary Data 4). We next performed Gene Ontology (GO) term analysis on our MDSC signature genes (Fig. 1L) using the Database for Annotation, Visualization and Integrated Discovery (DAVID). The top GO terms included “immune system process”, “inflammatory response”, “innate immune response”, “positive regulation of I-kappaB kinase/NF-kappaB signaling”, “negative regulation of T cell proliferation”, “negative regulation of inflammatory response”, “negative regulation of CD4-positive, alpha-beta T cell proliferation”, “positive regulation of regulatory T cell differentiation”. These terms included genes encoding CD300 family members (Cd300lb, Cd300ld, Cd300lf and Cd300c), which modulate a broad and diverse array of immune cell processes via their paired activating and inhibitory receptor functions^20^. Notably, Cd300ld has been reported as a marker of PMN-MDSCs in multiple tumor types, which induced immune suppression^17^. In addition, several moleculars proven to be essential for the expansion, activation, and immunosuppressive function of mouse and human MDSCs such as Stat3 and MyD88 were also included^21^. It is worth noting that Cd84 and Jaml also appeared on our MDSC signature gene list, which have been reported as markers of MDSCs from breast cancer^16^. Previous studies have additionally demonstrated that the expansion of MDSCs in the bone marrow of aged mice occurred via an NF-κB-mediated mechanism^9^. In the present study, the GO term “positive regulation of I-kappaB kinase/NF-kappaB signaling” encompasses genes such as Trim12c, Map3k3, Nfat5, Trim30b, and Clec4d, which potentially contribute to the expansion of MDSCs. We also conducted RT-qPCR to validate the expression levels of some reported MDSC signature genes, such as Arg2, Ifitm1, Il1b, Retnlg, Jaml, Clec4e et al., and found that they were significantly up-regulated in CD11b^+^Gr1^+^ cells from aged mice when compared with young mice (Fig. 1M).

### Identification of bone marrow-derived MDSCs in aged human using MDSC signature genes

**Fig. 2:**
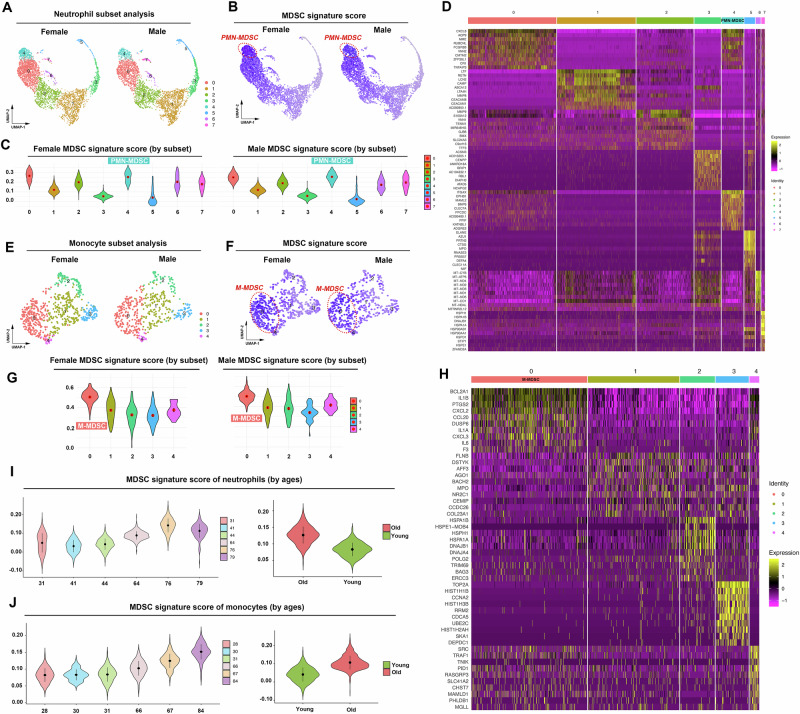
Identification of bone marrow-derived MDSCs in aged human using MDSC signature genes. **A** Unbiased Seurat clustering analysis of bone marrow-derived neutrophils in the integrated scRNAseq dataset. The UMAP projection result showed eight distinct clusters in the neutrophils, both in male and female samples. **B**, **C** UMAP and violin plot results showed MDSC signature score ordered by neutrophil subset. Cluster 4 exhibited the highest expression of MDSC gene signature, both in male and female samples. **D** Heat map showing top 10 marker genes for each neutrophil subset. **E** Unbiased Seurat clustering analysis of bone marrow derived monocytes in the integrated scRNAseq dataset. The UMAP projection result showed five distinct clusters in the monocytes, both in male and female samples. **F**, **G** UMAP and violin plot results showed MDSC signature score ordered by monocyte subset. Cluster 0 exhibited the highest expression of MDSC gene signature, both in male and female samples. **H** Heat map showing top 10 marker genes for each monocyte subset. **I** MDSC signature score escalated with advancing age in neutrophils. **J** MDSC signature score escalated with advancing age in monocytes.

To comprehensively assess the universal applicability of our MDSC signature genes in the context of human aging, we utilized publicly available scRNAseq datasets that include myeloid cells of aged males and females (with 9 myeloid cell subclusters) to conduct a validation (Fig. S5). However, the expressions of MDSCs signature genes in neutrophils and monocytes displayed a dispersed or non-specific pattern (Fig. S5), we then extracted the neutrophilic or monocytic lineage respectively, and reconducted the clustering analysis. Initially, we performed scRNAseq analysis on neutrophils isolated from the myeloid cells of healthy aged individuals. By employing an unbiased clustering approach, we identified eight distinct subsets of the neutrophils (Fig. 2A, D). Subsequently, we applied our MDSC gene signature in a gene scoring analysis to these neutrophil subsets, revealing that Cluster 4 exhibited the highest score, both in male and female samples (Fig. 2B, C). This finding underscored the efficacy of our signature genes in discerning PMN-MDSCs from their normal lineage counterparts in human. Subsequently, to ascertain the capacity of our MDSC gene signature in accurately differentiating M-MDSCs from normal monocytes, we analyzed scRNAseq data of monocytes isolated from the myeloid cells of healthy male and female individuals (Fig. 2E, H). Similarly, we found that Cluster 0 of monocyte exhibited the highest MDSC signature score (Fig. 2F, G). In summary, our findings revealed that the MDSC gene signature derived from the aged mouse model exhibited good compatibility in human aging systems, thereby suggesting the potential for a comparable MDSC state between mouse and human. Interestingly, analysis of single-cell datasets from individuals of different ages showed that the MDSC signature score increases with age in both neutrophils and monocytes (Fig. 2I, J), which further validated our findings.

### Identification of a specific cell surface marker for MDSCs in aged individuals

Given that our scRNAseq data have identified the signature genes of MDSCs during aging, we scrutinized these 472 genes in order to select the specific cell surface marker for MDSC detection and isolation. First, we used UniPort (https://www.uniprot.org/) to screen membrane-expressed proteins corresponding to the signature genes, and finally 119 signature genes were included (Supplementary Data 5). Among the 119 genes, we identified MDSC marker genes that have been previously reported in literature, such as Jaml, Ifitm1, Clec4d, Cxcr2, C5ar1, Cd33, Cd44^16,22^. Intriguingly, several Cd300 family members (Cd300lb, Cd300ld, Cd300lf, and CD300c) were also identified as potential surface markers, and Cd300ld has been previously reported as a surface marker of PMN-MDSCs in cancerous conditions^17^.

**Fig. 3:**
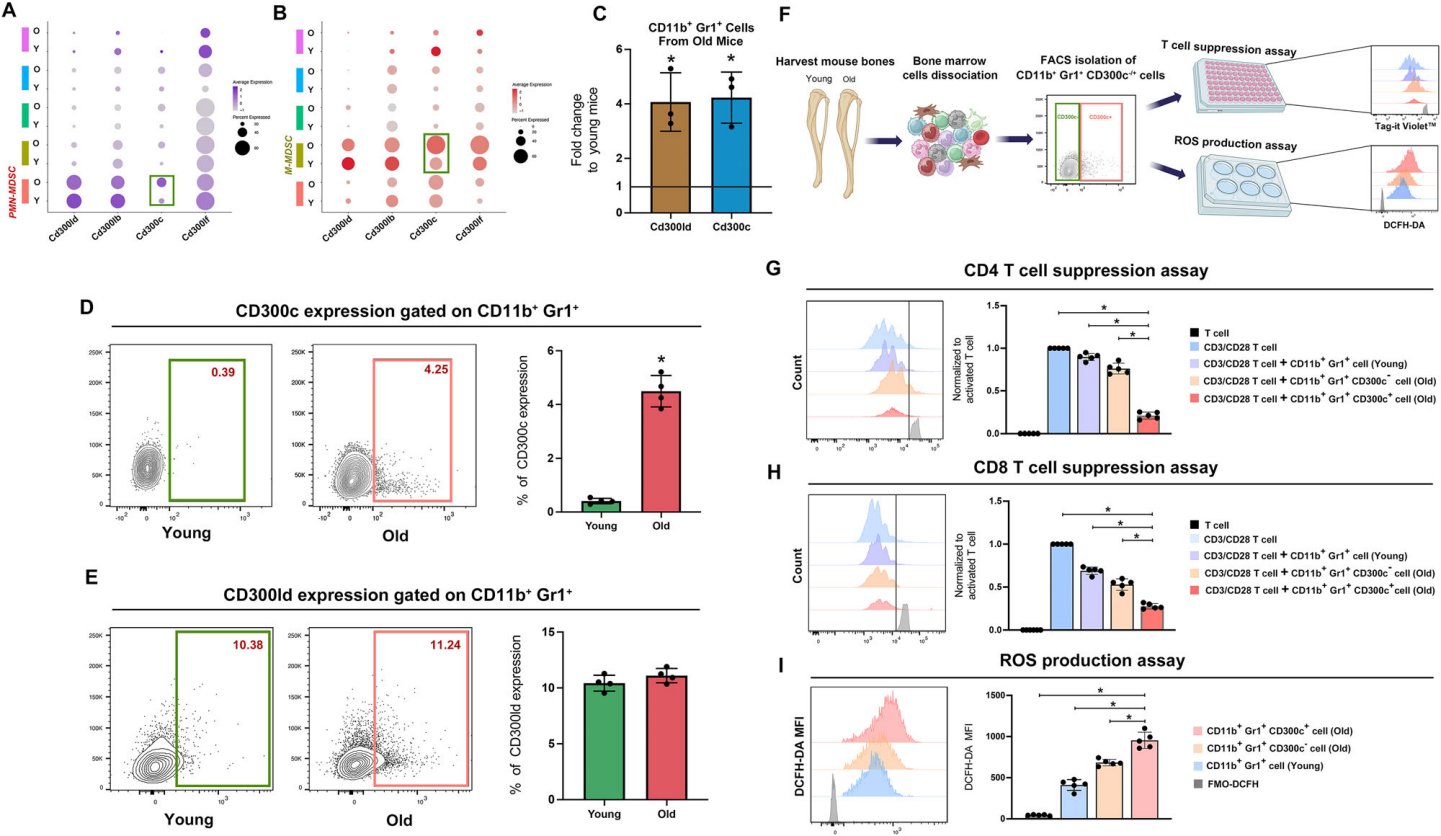
CD11b^+^Gr1^+^CD300c^+^ cells demonstrated a robust ability of T cell suppression and enhanced production of ROS. **A**, **B** Dot plots demonstrating the expression of Cd300 family members in different cell clusters in aged or young mice. The expression levels and percentages of Cd300c showed the most significant differences between aged and young mice in both PMN-MDSCs and M-MDSCs. **C** RT-qPCR result showed that Cd300ld and Cd300c were significantly up-regulated in aged mice, when compared to their young counterparts. **D** CD11b^+^Gr1^+^ cells from bone marrow of aged mice exhibited significantly high expression of CD300c when compared with their counterparts in young mice. **E** No significant difference of CD300ld was detected between CD11b^+^Gr1^+^ cells isolated from the bone marrow of young and aged mice. **F** Experimental design schematic. **G**, **H** T cell suppression assay results showed that CD11b^+^Gr1^+^CD300c^+^ cells isolated from the bone marrow of aged mice suppressed CD4 and CD8 T cell proliferation significantly, compared with CD11b^+^Gr1^+^ cells from young mice and CD11b^+^Gr1^+^CD300c^-^ cells from aged mice. **I** CD11b^+^Gr1^+^CD300c^+^ cells isolated from the bone marrow of aged mice showed increased ROS production compared with CD11b^+^Gr1^+^ cells from young mice and CD11b^+^Gr1^+^CD300c^-^ cells from aged mice. Data were presented as mean ± SD. *P < 0.05.

We next analyzed and compared the expression levels of the 119 genes in PMN-MDSCs or M-MDSCs between aged and young mice, respectively (Fig. S6, Fig. 3A, B). The dot plot results showed that the expression level and percentage of Cd300c showed the most significant differences between aged and young mice, both in PMN-MDSCs and M-MDSCs, which was never reported before (Fig. 3A, B). RT-qPCR result showed that Cd300ld and Cd300c were significantly up-regulated in CD11b^+^Gr1^+^ cells of aged mice, when compared to their young counterparts (Fig. 3C). We then conducted FACS to examine the percentage of CD300c^+^ cells in CD11b^+^Gr1^+^ myeloid cells in the bone marrow of young and aged mice, and found that the CD11b^+^Gr1^+^ cells from bone marrow of aged mice exhibited significantly higher expression of CD300c than young controls (Fig. 3D). While no significant difference was observed in the previously reported CD300ld between young and aged mice (Fig. 3E). Therefore, we surmised that CD300c may serve as a distinctive cell surface marker for MDSCs during aging process.

### CD300c^+^ MDSCs exhibited a robust capacity for T cell suppression

As CD300c has been found to exhibit significant differential expression in myeloid cells between aged and young mice, which has not been reported previously, we hypothesize that CD300c may emerge as a specific surface marker of MDSCs in aging process. However, whether the CD11b^+^Gr1^+^CD300c^+^ cells could possess potent T cell suppression function and product MDSC-related immunosuppressive factors such as ROS still remains unknown.

Firstly, we performed cocultures with activated T cells as described in Fig. 3F. The cell proliferation dye results showed that CD11b^+^Gr1^+^CD300c^+^ cells from bone marrow of aged mice suppressed both CD4 and CD8 T cell proliferation significantly, whereas CD11b^+^Gr1^+^CD300^-^ cells in aged mice displayed a weaker inhibitory impact (Fig. 3G-H). Next, we used 2,7-Dichlorodihydrofluorescein diacetate (DCFH-DA) to detect ROS production in CD11b^+^Gr1^+^CD300c^+^ cells. As shown in Fig. 3I, CD11b^+^Gr1^+^CD300c^+^ cells produced highest amounts of ROS compared with CD11b^+^Gr1^+^ cells from young mice and CD11b^+^Gr1^+^CD300c^−^ cells from aged mice. These findings revealed that CD300c may serve as a specific marker of MDSCs during aging and mediate powerful immunosuppression function.

### MDSCs from bone marrow of aged mouse share the similar developmental trajectory with neutrophilic or monocytic lineage, and may develop from mature myeloid cells

It is generally recognized that MDSCs are characterized by their myeloid origin, immature state, and immunosuppressive ability^7^, and play crucial roles in the pathogenesis of cancers, chronic infections and autoimmune diseases^7,12^. Previous study has reported that MDSCs emerge through an aberrant differentiation trajectory during breast cancer^16^, however whether the process still exists during physiological aging remains elusive.

**Fig. 4:**
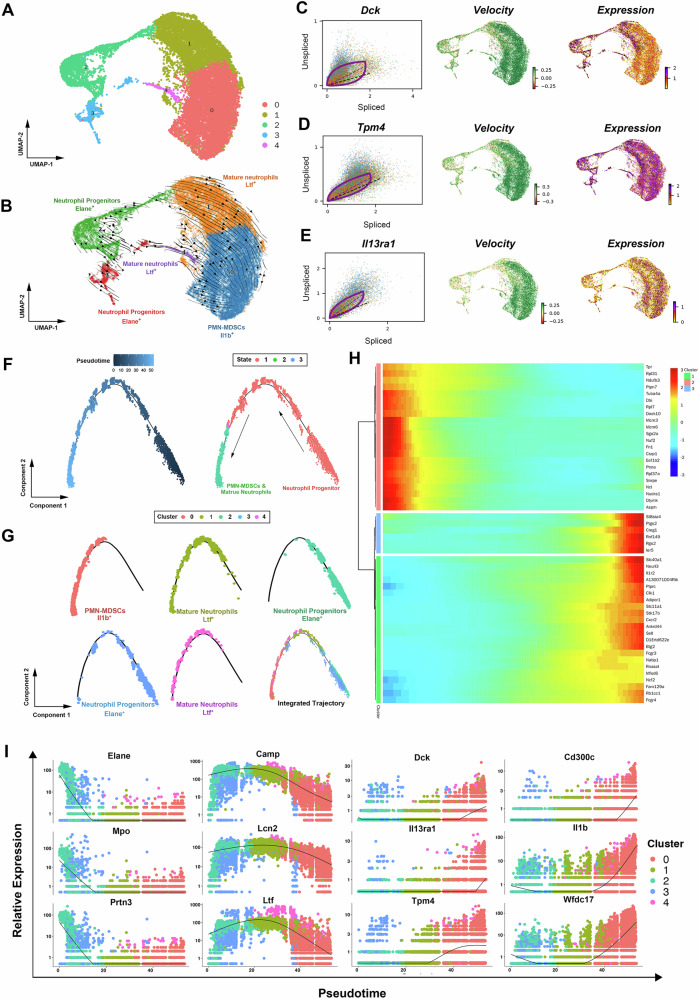
PMN-MDSCs from bone marrow share the similar developmental trajectory with neutrophilic lineage during aging, and may partially develop from mature neutrophils. **A**, **B** Velocities derived from the dynamical model for PMN-MDSC generation are projected into a UMAP based embedding. Three types of cells were identified according to previously reported marker genes. Including neutrophil progenitors (Elane, Mpo, Prtn3), mature neutrophils (Camp, Ltf, and Lcn2), and PMN-MDSCs (Cd300c, Il1b, Wfdc17). The gene-averaged flow of neutrophil lineage visualized by velocity streamlines showed that neutrophil progenitors developed into mature neutrophils and then PMN-MDSCs. **C–E** Dynamical model was used to identify driver genes in PMN-MDSC generation. **F** Developmental trajectory of neutrophil subsets by pseudotime value. **G** Distribution of five neutrophil subsets along the developmental trajectory. PMN-MDSCs had the highest pseudotime value and were located at the ending point, whereas the neutrophil progenitors had the lowest pseudotime value and were located at the starting point. **H** Clustered heat map revealing top 50 genes with the most significant alterations across pseudotime in neutrophil population. **I** Pseudotime plot illustrating expression of selected marker genes over pseudotime. The neutrophil progenitor markers (Elane, Mpo, Prtn3) exhibited a declining trend across the pseudotime trajectory, the mature neutrophil markers (Camp, Ltf, and Lcn2) demonstrated a distinctive “rising first and then falling” pattern, while PMN-MDSC marker genes (Cd300c, Il1b, Wfdc17) and driver genes (Dck, Tpm4, and Il13ra1) showed an increased trend.

To explore the process of PMN-MDSC generation in the bone marrow, we first performed RNA velocity analysis. As shown in Fig. 4A-B, after basic pre-processing, we applied the dynamical model and displayed the vector field using streamline plots in a uniform manifold approximation and projection (UMAP)-based embedding. Three subsets were identified according to previously reported marker genes. Neutrophil progenitors are marked by the genes Elane, Mpo, and Prtn3^23^. Mature neutrophils, on the other hand, exhibit elevated levels of Camp, Ltf, and Lcn2^23^. PMN-MDSCs, as revealed in the current study, are characterized by the markers Cd300c, Il1b, and Wfdc17. The gene-averaged flow of neutrophil lineage visualized by velocity streamlines showed that neutrophil progenitors differentiated into mature neutrophils and then PMN-MDSCs, which was different from former study (Fig. 4B)^16^. In addition, the dynamical model allowed to systematically identify driver genes with high likelihoods in PMN-MDSC generation, top likelihood-ranked genes included Dck, Tpm4, and Il13ra1 (Fig. 4C-E). Next, in order to further confirm the results of RNA velocity, we performed Monocle for unsupervised pseudotemporal ordering of neutrophil subsets. Which resulted in a trajectory with almost no branch and three distinct states (Fig. 4F-G). In combination with the cell identification results, we depicted the development trajectory of PMN-MDSCs. As shown in Fig. 4F-G, neutrophil progenitors were located at the beginning of pseudotime (Cluster 3 and Cluster 2), while the developmental trajectories of PMN-MDSCs (Cluster 0) and mature neutrophils (Cluster 1 and Cluster 4) exhibited an overlap. Further analysis revealed that PMN-MDSCs showed a tendency to concentrate more toward the end of developmental trajectory than mature neutrophils. This finding indicated that in the bone marrow of aged mouse, PMN-MDSCs may generated from mature neutrophils, which was reported by a limited number of studies. In addition, the top 50 genes with significant changes over pseudotime were clustered, many genes related to immunosuppression showed an increased pattern, such as St8sia4, Ptgs2, and Il1r2 (Fig. 4H). Gene plots over pseudotime were also analyzed to further confirm our findings. The neutrophil progenitor markers (Elane, Mpo, Prtn3) exhibited a declining trend across the pseudotime trajectory, the mature neutrophil markers (Camp, Ltf, and Lcn2) demonstrated a distinctive “rising first and then falling” pattern, while PMN-MDSC marker genes (Cd300c, Il1b, Wfdc17) and PMN-MDSC driver genes (Dck, Tpm4, and Il13ra1) showed an increased trend (Fig. 4I).

**Fig. 5:**
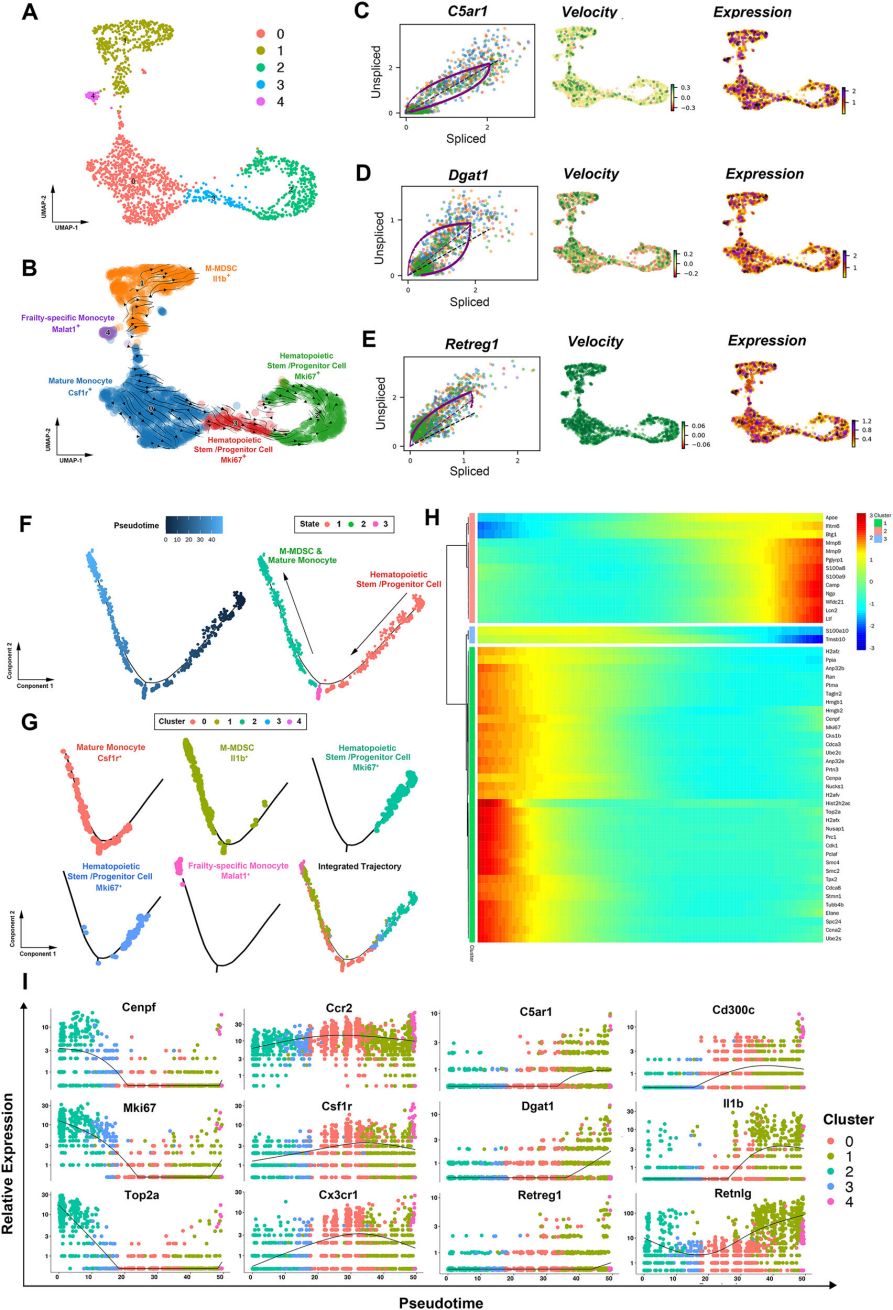
M-MDSCs from bone marrow share a similar developmental trajectory with monocytic lineage during aging, and may partially develop from mature monocytes. **A**, **B** Velocities derived from the dynamical model for M-MDSC generation are projected into a UMAP based embedding. Four types of cells were identified according to previously reported marker genes. Including hematopoietic stem/progenitor cells (Cenpf, Mki67, and Top2a), mature monocytes (Ccr2, Csf1r, Cx3cr1), frailty-specific monocytes (Malat1, Neat1) and PMN-MDSCs (Cd300c, Il1b, Retnlg). **C–E** Dynamical model was used to identify driver genes in M-MDSC generation. **F** Developmental trajectory of monocyte subsets by pseudotime value. **G** Distribution of five monocyte subsets along the developmental trajectory. M-MDSCs had the highest pseudotime value and were located at the ending point, whereas the hematopoietic stem/progenitor cells (HSPCs) had the lowest pseudotime value and were located at the starting point. **H** Clustered heatmap revealing top 50 genes with the most significant alterations across pseudotime in monocyte population. **I** Pseudotime plot illustrating expression of selected marker genes over pseudotime. The hematopoietic stem/progenitor cell markers (Cenpf, Mki67, Top2a) exhibited a declining trend across the pseudotime trajectory, the mature monocyte markers (Ccr2, Csf1r, Cx3cr1) demonstrated a “rising first and then falling” pattern, while M-MDSC marker genes (Cd300c, Il1b, Retnlg) and driver genes (C5ar1, Dgat1, Retreg1) showed an increased trend.

Next, we applied RNA velocity and Monocle analyses to explore the developmental trajectory of M-MDSCs. Four subsets were identified according to previously reported marker genes. Hematopoietic stem/progenitor cells (HSPCs) are marked by the genes Cenpf, Mki67, and Top2a^24,25^. Mature monocytes exhibit elevated levels of Ccr2, Csf1r, and Cx3cr1^16,26^. M-MDSCs, as reported in the current study, are characterized by the markers Cd300c, Il1b, and Retnlg. In addition, a small group of cells are identified as frailty-specific monocytes by the markers Malat1 and Neat1^27^. The streamline plot of a UMAP-based embedding showed non-obvious linear-like developmental trajectory (Fig. 5A, B). Dynamical model showed driver genes with high likelihoods in M-MDSC generation, top likelihood-ranked genes included C5ar1, Dgat1, and Retreg1 (Fig. 5C-E). In addition, Monocle analysis revealed the developmental trajectory of M-MDSCs similar with PMN-MDSCs. In brief, M-MDSCs originate from HSPCs (Cluster 2 and Cluster 3), and shared a similar developmental trajectory with mature monocytes. It was also shown that M-MDSCs (Cluster 1) exhibited a greater tendency to concentrate towards the end of developmental trajectory, when compared to mature monocytes (Cluster 0) (Fig. 5F, G). Top 50 genes with significant changes over pseudotime also included genes related to immunosuppression, such as S100a8, S100a9, and Pglyrp1 (Fig. 5H). Gene plots over pseudotime of HSPC markers (Cenpf, Mki67, and Top2a), mature monocyte markers (Ccr2, Csf1r, Cx3cr1), M-MDSC markers (Cd300c, Il1b, Retnlg) and M-MDSC driver genes (C5ar1, Dgat1, Retreg1) showed similar trends with PMN-MDSCs (Fig. 5I).

### The developmental trajectory of MDSCs in the bone marrow of elderly humans is consistent with that of aged mice

**Fig. 6:**
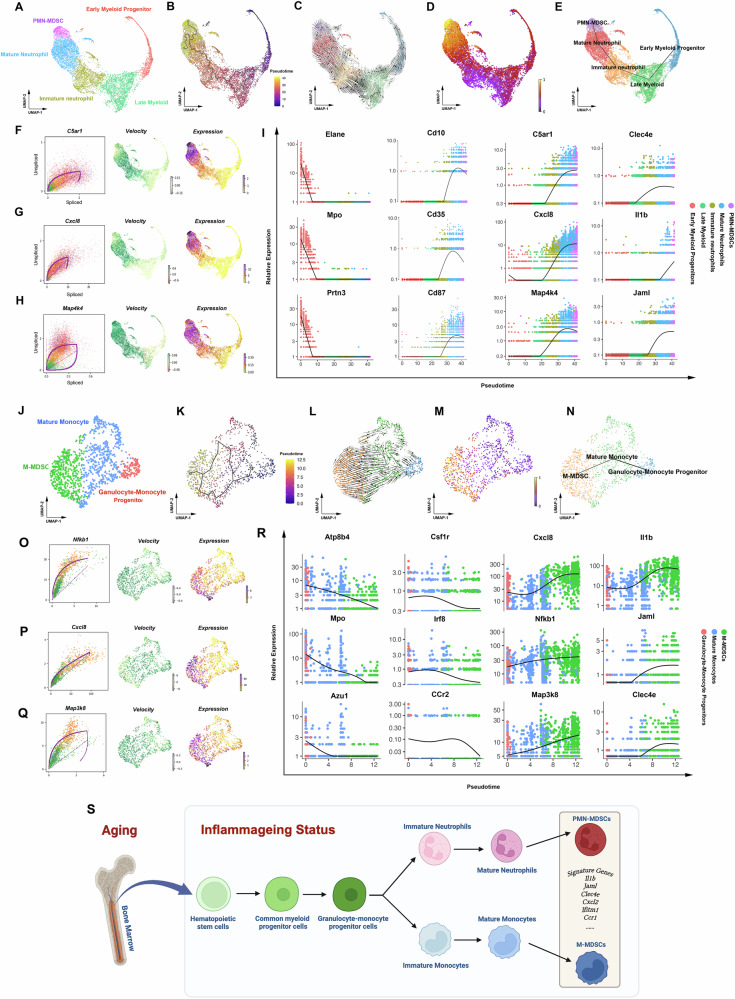
The developmental trajectory of MDSCs in the bone marrow of elderly humans is consistent with that of aged mice. **A** Five types of cells were identified, mainly including early myeloid progenitors, late myeloid, immature neutrophils, mature neutrophils, and PMN-MDSCs. **B** The single-cell trajectory of neutrophilic cells was predicted by Monocle 3 and visualized by UMAP. Cells are ordered in pseudotime colored in a gradient from purple to yellow. **C-E** The gene-averaged flow of neutrophil lineage visualized by velocity streamlines showed that neutrophil progenitors developed into mature neutrophils and then PMN-MDSCs. **F-H** Dynamical model was used to identify driver genes in PMN-MDSC generation. **I** Pseudotime plot illustrating expression of selected marker genes over pseudotime. The neutrophil progenitor markers (Elane, Mpo, Prtn3) exhibited a declining trend across the pseudotime trajectory, the mature neutrophil markers (Cd10, Cd35, Cd87) demonstrated a distinctive “rising first and then falling” pattern, while PMN-MDSC marker genes (Clec4e, Il1b, Jaml) and driver genes (C5ar1, Cxcl8, and Map4k4) showed an increased trend. **J** Three types of cells were identified according to previously reported marker genes. Including granulocyte-monocyte progenitors, mature monocytes, and M-MDSCs. **K** The single-cell trajectory of monocytic cells was predicted by Monocle 3 and visualized by UMAP. Cells are ordered in pseudotime colored in a gradient from purple to yellow. **L–N** The gene-averaged flow of monocyte lineage visualized by velocity streamlines showed that GMP developed into mature monocytes and then M-MDSCs. **O-Q** Dynamical model was used to identify driver genes in M-MDSC generation. **R** Pseudotime plot illustrating expression of selected marker genes over pseudotime. The GMP markers (Atp8b4, Mpo, Azu1) exhibited a declining trend across the pseudotime trajectory, the mature monocyte markers (Csf1r, Irf8, and Ccr2) demonstrated a distinctive “rising first and then falling” pattern, while M-MDSC marker genes (Clec4e, Il1b, Jaml) and driver genes (Nfkb1, Cxcl8, and Map3k8) showed an increased trend. **S** MDSCs may develop from mature myeloid cells during the aging process, this developmental trajectory is distinctly different from that observed in cancer.

To verify whether the developmental trajectory of MDSCs in mouse bone marrow also applies to human, we conducted Monocle3 and RNA velocity analyses on the single-cell datasets derived from neutrophilic or monocytic cells in elderly individuals as mentioned previously. As shown in Fig. 6A-E, both Monocle3 and RNA velocity results revealed that PMN-MDSC may develop from mature neutrophils. In addition, the neutrophil progenitor markers (Elane, Mpo, Prtn3), mature neutrophil markers (Cd10, Cd35, Cd87)^28^, MDSC marker genes (Clec4e, Il1b, Jaml) and PMN-MDSC driver genes (C5ar1, Cxcl8, Map4k4) showed similar trends with aged mice (Fig. 6F-I). Our research also found that the developmental trajectory of human M-MDSCs in the elderly is similar to that in aged mice, indicating that M-MDSCs may originate from mature monocytes (Fig. 6J-N). Furthermore, gene plots over pseudotime of granulocyte-monocyte progenitor markers (Atp8b4, Mpo, Azu1), mature monocyte markers (Csf1r, Irf8, Ccr2), M-MDSC driver genes (Cxcl8, Nfkb1, Map3k8) and MDSC marker genes (Clec4e, Il1b, Jaml) demonstrated a similar trend of change as observed in aged mice (Fig. 6O-R).

In conclusion, based on the results of single-cell sequencing data from both mice and humans, we believe that MDSCs can develop from mature myeloid cells during the aging process (Fig. 6S). This developmental trajectory is distinctly different from that observed in cancer and has been reported by a limited number of studies^29–32^.

## Discussion

Inflammageing is characterized by a persistent low-grade inflammation in the elderly, which is ubiquitous in the aging process^3^. It is now known that inflammaging is linked to augmented myelopoiesis in the bone marrow, which results in the amplified production of not only mature myeloid cells, such as neutrophils, monocytes and macrophages, but also MDSCs^4,33^. The generated MDSCs are then recruited to inflammatory tissues and suppress the inflammatory responses^4,12^. However, if the chronic inflammation state could not be alleviated, the long-term presence of MDSCs will suppress the immune system in aged individuals, thereby inducing immunosenescence and increasing elderly people’s susceptibility to infections, cancers, autoimmune diseases, and degenerative diseases^12,34–36^. Even worse, the proliferation of immunosuppressive MDSCs has the potential to exacerbate chronic inflammation and intensify immunosenescence, thereby fostering a vicious cycle, which underscores the crucial role of MDSCs in sustaining immunosenescence^12^.

It is now known that MDSCs comprise a heterogeneous group of immunosuppressive myeloid cells originating from the common myeloid progenitors^5^. However, the most commonly used MDSC markers (both mouse and human) overlap with those defining healthy and normal neutrophils or monocytes, which makes it challenging to distinguish MDSCs from their myeloid counterparts^15^. This will hamper the deeper understanding of the pathophysiological functions of MDSCs. In recent years, scholars attempted to identify the phenotypes or specific markers of MDSCs, and found that the phenotypes of MDSCs may be distinct in different pathological or physiological states. For instance, Gabrilovich et al. found that Il1b and Arg2 were two MDSC-related genes which could mediate immunosuppressive function^13^. Condamine et al. found that LOX was a specific marker of human PMN-MDSCs that can be used to identify these cells in the blood and tumors of patients with cancer^18^. Alshetaiwi et al. identified CD84 as a surface marker of MDSCs in breast cancer^16^. Nevertheless, to date there still has no research specifically addressing the phenotyping of MDSCs in aged individuals. Hence, we conducted a scRNAseq to describe the gene signature of MDSCs during aging process and to elucidate how these immunosuppressive cells differ from normal neutrophils or monocytes.

In the current study, CD11b^+^Gr1^+^ myeloid cells were isolated from the bone marrow of both young and aged mice by FACS. Subsequently, we employed the well-documented immunosuppressive factors of MDSCs, Arg2, Il1b, Cd84, Wfdc17, Jaml, Stat3 and Cd33, to preliminarily identify PMN-MDSCs from neutrophils and M-MDSCs from monocytes by scRNAseq, respectively. It is noteworthy to observe a significant marker gene overlap between PMN-MDSCs and M-MDSCs, encompassing a total of 472 genes. The GO term analysis of the 472 genes mainly included the biological process of immune and inflammatory responses, inhibition of T cell proliferation, and induction of Tregs, which aligned well with the documented pathological features of MDSCs. Additionally, the GO term encompassed NF-κB activation, a finding that is congruent with prior research demonstrating that NF-κB is activated with aging^9^, thereby contributing to an elevated percentage of MDSCs in the bone marrow. Surprisingly, our signature gene list could also effectively distinguish human MDSCs from normal myeloid cells, and the overall expression level of these signature genes gradually increases with aging. Based on the aforementioned results, we believe that the 472 overlapping genes have enabled us to establish distinctive gene signatures for MDSCs, thereby elucidating the transcriptome characteristics of bone marrow-derived MDSCs during aging.

A deeper analysis of the signature gene list revealed the presence of C5ar1, Cxcr2, Csf3r, and Ccr1, which play pivotal roles in the recruitment, accumulation, and trafficking of MDSCs^37–39^. This revelation underscored the potential of MDSCs to respond to inflammatory cues, thereby directing their migration towards sites of inflammation. Additionally, genes involved in immunosuppression, such as Wfdc17^40^, and Myd88^41^, were also identified within the signature gene list. Notably, we also found the inclusion of previously reported MDSC marker genes, such as Cd300ld, Cd33, Cd84, Jaml, Clec4d, Clec4e, Junb, and Ctsd^16^. These findings validate that MDSCs derived from bone marrow during the aging process share certain gene signatures with MDSCs found in other pathological states.

We performed RNA velocity and pseudotime analysis to elucidate the emergence of bone marrow-derived MDSCs during aging. Previous studies reported that both PMN-MDSCs and M-MDSCs originate from neutrophil or monocyte progenitors via an aberrant differentiation trajectory respectively, leading to a cell state that is not present in normal conditions^16^. However, the cell development analysis results from both mouse and human myeloid cells indicated that the situation in healthy older individuals significantly differs from the previous findings. Firstly, the pseudotime trajectory of cell development exhibited no discernible branching, both in PMN-MDSCs and M-MDSCs, which suggested a congruency in the developmental trajectory between MDSCs and mature myeloid cells. Secondly, both PMN-MDSCs and M-MDSCs showed a tendency to concentrate more toward the end of developmental trajectories than their mature myeloid counterparts, which indicated that MDSCs from aged individual may develop from mature neutrophils or monocytes. This is a significant departure from the traditional understanding that MDSCs are immature myeloid cells derived from myeloid progenitors. And our findings have also been reported by several studies, in which the terminal or more differentiated myeloid cells can “move backwards” to MDSCs^29–32^. Recently, researchers also demonstrated that the maturity of cells is largely irrelevant for the MDSC definition, the maturation state depends on the strength of signals that MDSCs receive, which vary between different status^42^. In our opinion, this divergence could stem from the fundamental differences between natural aging, a normal physiological process, and pathological conditions like tumors or inflammation. Consequently, this may lead to variations in the differentiation trajectory of MDSCs, one of the characteristics that distinguishes MDSCs in elderly individuals from those observed in other pathological states.

Literature has reported early-stage MDSCs (E-MDSCs) are the least mature myeloid-derived suppressor cells, identified only in humans and characterized by the phenotype CD3⁻CD14⁻CD15⁻CD19⁻CD56⁻HLA-DR⁻CD33⁺^6^. Generally, E-MDSCs were considered as the progenitor of both PMN-MDSCs and M-MDSCs. Thus, we analyzed the single-cell transcriptomic data which profiles human myeloid cells of aged individuals. This dataset revealed nine distinct myeloid cell clusters. However, no subpopulation simultaneously exhibited the CD3⁻CD14⁻CD15⁻CD19⁻CD56⁻HLA-DR⁻CD33⁺ signature (Fig. S7). Therefore, we consider that the E-MDSCs may not exist in the healthy elderly population, and integrating our current findings, we propose that M-MDSCs or PMN-MDSCs may arise from their mature myeloid counterparts instead of E-MDSCs.

In humans, there have been some new classifications for myeloid cells. Which included MS1 cells, low density neutrophils (LDNs) and exhausted monocytes, as the transcriptome of these can be very similar with MDSCs. Therefore, we also investigated whether these cells are present in inflammaging process. MS1 cells were CD14^+^ cells characterized by high expression of resistin (RETN), arachidonate 5-lipoxygenase activating protein (ALOX5AP) and interleukin-1 receptor type 2 (IL1R2)^43^. Reyes et al. reported top 15 MS1 marker genes to identify these cells^43^. We applied “AUCell” to score the characteristic gene set of MS1 cells, but found that these methods could not identify MS1 cells in healthy aged human monocytic lineages (Fig. S8). Reyes et al. also reported that BMMCs treated with LPS or Pam3CSK4 revealed a cluster of cells scoring highly for MS1 signature genes that are absent in the untreated conditions^43^. Therefore, we consider that MS1 cells may not exist in the healthy elderly population. LDNs are defined as an unique subpopulation of neutrophils, and reported to play a significant role in regulating innate and adaptive immunity in various inflammation-related diseases^44,45^. Common markers used for LDNs identification include CD15, CD16, and CD66b, in addition, CD10, CD11b, CD33, CD35, and CD63, also enables further functional characterization^44^. We then visualized the marker genes of LDNs in the aged human neutrophil dataset and found their expression in both cluster 0 and cluster 4, with more prominent expression in cluster 0. Given that we have previously identified cluster 4 as PMN-MDSCs, we performed differential gene expression analysis (volcano plot) between cluster 0 and cluster 4 (Fig. S9). The reported LDN markers CD10 and CD35 both showed significant differences. This suggests that in healthy elderly individuals, PMN-MDSCs and LDNs may not represent the same cell population. We posit that in healthy aged individuals, LDNs are likely intermingled with mature neutrophils and lack a distinct functional role, a perspective consistent with the earlier report by Hardisty et al.^45^. Key features of monocyte exhaustion, including reduced differentiation, pathogenic inflammation and immune suppression which is similar with M-MDSCs^46–48^. Literatures reported that exhausted monocytes are characterized by the molecular signature MKI67^hi^ CD38^hi^ CD86^low^HLA-DRA^low^, which corresponds to their less mature, pathogenic inflammatory, and immunosuppressive properties, respectively^47^. So we tried to identify exhausted monocytes in aged human monocyte dataset, however, no monocyte subpopulation simultaneously exhibits the MKI67^hi^ CD38^hi^ CD86^low^HLA-DRA^low^ signature (Fig. S10). Indeed, studies indicate that exhausted monocytes emerge primarily in response to severe sepsis or prolonged, high-dose LPS exposure^46,47^. In contrast, this specific cell population appears to be absent in the context of low-grade chronic inflammation, such as inflammaging. Thus, we consider that exhausted monocytes might not be present in the healthy elderly population. In summary, we propose that the aforementioned cells (MS1 cells, LNDs, exhausted monocytes) may be present in acute inflammatory responses or sepsis, but are likely absent in the low-grade chronic inflammation characteristic of inflammaging.

As far as we know, there is no report on the specific marker of MDSCs in aged individuals, which hinders further research into the functions of MDSCs. In the present study we found that CD300c may serve as the specific marker of MDSCs during aging. Even though the proportion of CD11b^+^Gr1^+^CD300c^+^ cells in the bone marrow of aged mice is relatively low, there is still a significant increase of over tenfold compared to young counterparts. The CD11b^+^Gr1^+^CD300c^+^ cells also showed a strong ability of CD4 and CD8 T cell suppression and ROS production. CD300c was originally isolated from human leukocytes, and also known as CMRF-35A or CMRF-35^49^. It was reported that CD300c may affect monocytes, DCs, macrophages, NK cells, basophils and B cells. In 2018, Cui et al. found CD300c as a novel T cell inhibitory molecule, both soluble human and mouse CD300c-Fc fusion proteins significantly inhibit the proliferation, activation, and cytokine production of CD4 and CD8 T cells in vitro^50^. In recent year, CD300c-Ig was found to decrease the expansion and activation of T cells and reduce expression of proinflammatory cytokines in the mouse model of collagen-induced arthritis^51^. CD300ld was once reported as a critical immune suppressor present on PMN-MDSCs^17^, although CD300c and CD300ld are genomically adjacent, their expression patterns and functions in immune cells are markedly different, suggesting distinct regulatory mechanisms and biological roles. In line with Wang et al., who reported CD300ld is constitutively expressed in normal neutrophils and specifically upregulated in PMN-MDSCs upon tumorigenesis^17^, our findings demonstrate that CD300ld expression remains statistically unchanged in myeloid cells during healthy aging. This consistency reinforces the concept that CD300ld dysregulation is a cancer-associated phenomenon rather than an age-related one. In addition, research on CD300c is relatively limited, with literature reporting its roles primarily in inflammation, degenerative diseases, and allergies^51–53^. Briefly, although CD300ld and CD300c are genomically adjacent, we believe they mediate immune regulation in different states. In combination with our findings, we consider that CD300c mediate the immunosuppression function of MDSCs, which may serve as a specific surface marker for MDSCs identification during aging process.

There are some limitations in the present study. First, while we have characterized the gene signature and specific surface marker of MDSCs during aging and demonstrated their immunosuppressive function in vitro, it is necessary to conduct in vivo assays to further validate the functional significance of these cells. Therefore, using CD300c-knockout aged mice as a model for the next stage of research may constitute a future direction. Second, sexual dimorphism significantly influences leukocyte differentiation and immune responses, leading to distinct immunological profiles between males and females. At the cellular level, sex differences are observed in the proportions and functions of various leukocyte subsets. For instance, males often have higher levels of neutrophils, while females tend to have an increased proportion of lymphocytes^54^. In the present study, male mice were used in our scRNAseq process because male animals exhibited less variability in phenotype. To determine the role of sex, we assessed myeloid cell datasets from human donors of both sexes and identified MDSC subsets with comparable expression profiles in males and females. However, whether the immunosuppressive potency differs between sexes requires further investigation. Third, the transcriptional profiling of human neutrophils remains technically challenging due to their characteristically high RNase activity and low RNA content, which may compromise RNA integrity and sequencing depth. Although recent methodological progress—such as the 10X Genomics platform—has gradually made such assays more feasible, as reflected in a growing number of studies, these inherent limitations persist. It is within this context that we employed human neutrophil transcriptomes primarily as a means of cross-species validation for murine MDSC signatures, rather than as a source of quantitatively absolute gene expression data. Moreover, our analytical focus was placed on detecting relative transcriptional differences between age groups. We anticipate that future technical advances will be necessary to fully overcome these barriers and enable more comprehensive, high-resolution transcriptomic characterization of neutrophils.

In conclusion, we revealed the unique gene signature and specific surface marker of MDSCs during aging. And clarified that MDSCs from bone marrow of aged individuals share the similar developmental trajectory with normal myeloid lineage and may develop from mature myeloid cells. Our work extends the understanding of MDSCs in aging process.

## Methods

### Mouse bone marrow cells collection

All animal experiments conducted in this study were approved by Nanfang Hospital Animal Ethic Committee, Southern Medical University (Ethics Approval Number: NFYY-2020-0261). The mouse bone marrow cells were collected as previously described^35^. Briefly, three aged male mice (20 months old) and three young male mice (8 weeks old) were sacrificed by cervical dislocation. And then detached the femur from the tibia. Cut the proximal and distal ends of the bones, the bone marrow was flushed from mouse tibias and femurs using a 25 G ×5/8” needle and plastic syringe and then kept in phosphate buffer saline (PBS) with 5% fetal bovine serum (FBS) on ice. Bone marrow cells were centrifuged at 400 × *g* at 4 °C for 10 min. Resuspending the cells in 2 ml NH_4_Cl_2_ (pH: 7.2–7.6) for 5 min at room temperature to lyse the erythrocytes. Then centrifuged for 10 min, at 400 × *g*, at 4°C and resuspended the cells in 1 ml of 5% PBS/FBS and kept at 4 °C for the next step.

### Fluorescence-activated cell sorting (FACS)

Bone marrow samples collected from the mouse were centrifuged at 400 × *g* at 4 °C for 5 min and washed once with 5% PBS/FBS. Cells were blocked with anti-mouse CD16/32 (BioLegend, 101302) on ice for at least 10 min. Cells were then incubated for 30 min at 4 °C with pre-conjugated fluorescence-labeled antibodies with the following combinations: APC anti-mouse Ly-6G/Ly-6C (Gr-1) Antibody (BioLegend, 108412), FITC anti-mouse/human CD11b Antibody (BioLegend, 101206), PE anti-mouse CD300c/d Antibody (BioLegend, 148003). Fixable Viability Dye eFluor™ 450 (eBioscience, 65-0863) was added to stained cells to assay for viability. Cells were sorted with BD FACSAria™ III, and the selected populations were isolated for the next experiments.

### Quantitative reverse transcription polymerase chain reaction (RT-qPCR)

Cells were transferred to RNase/DNase-free tubes. Single-cell lysis solution (1 × 10^3^ cells with 4 µl lysis buffer) was added into tubes and then incubated at room temperature for 5 min. Following cDNA synthesis, reverse transcription was performed in a thermal cycler, and gene expression pre-amplification primers were mixed with pre-amplification reaction mix based on the kit instructions (95 °C for 10 min, 14 cycles of 95 °C for 15 s, 60 °C for 4 min). The products from the pre-amplification stage were used for the real-time PCR reaction. The primer pair sequences used for the qPCR are listed in Supplementary Data 6. qPCR was performed using SYBR® Premix Ex Taq™ II (Takara) with a LightCycler 480 (Roche). Results were reported relative to control cells after being normalized to GAPDH using the △△CT method.

### Single-cell RNA sequencing

CD11b^+^Gr1^+^ myeloid cells selected by FACS from bone marrow of aged and young mice were washed with 0.05% PBS/bovine serum albumin (BSA, Sigma-Aldrich), resuspended to the concentration of 1000 cells/μl, then loaded onto the 10X Genomics Chromium platform for droplet-enabled scRNAseq according to the manufacturer’s instructions. The libraries were then sequenced on HiSeq3000 (Illumina) and analyzed using Cell Ranger version 4.0.0 (10X Genomics). Cell Ranger Count was used to produce an aligned single-cell feature count matrix, using mm38 genome as a reference. The “Cell Ranger Aggr” function was employed to standardize gene expression counts, derived from Cell Ranger Count, to a uniform sequencing depth, thereby mitigating deviations. Subsequently, the characteristic barcoded single-cell gene expression matrix was recalculated and generated. To discard doublets or dead cells, cells with gene counts <500 or >5000, or a mitochondrial gene ratio > 20%, were filtered out. Finally, a total of 23,143 cells were sequenced for downstream bioinformatic analysis.

### Subset identification of mouse myeloid cells using Seurat

Seurat version 5.2 was used to perform dimensionality reduction and clustering of the data, and conduct cluster identification. Briefly, Seurat’s canonical correlation analysis (CCA) was used to integrate cells from aged and young mice. Uniform manifold approximation and projection (UMAP) was used to project the cells into 2D space. Marker genes for each cluster were identified via the “FindAllMarkers” function of Seurat. Classical myeloid cell markers, such as Ly6g and Cxcr2 for neutrophils, Csf1r and Ccr2 for monocytes, were used to identify different cell subsets.

### T cell suppression assay

Spleens were dissected from mouse aged 8 weeks, then homogenized the tissue with a syringe plunger to obtain single cell suspension, lysed the erythrocytes using Red Blood Cell Lysing Buffer Hybri-Max™ (Sigma-Aldrich, R7757). T cells were isolated by magnetic bead negative selection from the spleen using the EasySep™ Mouse T Cell Isolation Kit (STEMCELL Technologies, 19851) according to the manufacturer’s instructions. The isolated T cells were washed once with PBS and resuspended with 0.01% PBS/BSA (Sigma-Aldrich). In order to detect the proliferation of T cells, all the selected cells were stained with Tag-it Violet™ Proliferation and Cell Tracking Dye (Biolegend, 425101) at a concentration of 5 μM per 1 × 10^7^ cells and incubated at 37 °C for 20 min. Next, the stained T cells were washed and incubated with RMPI 1640 with 10% FBS at the concentration 1 × 10^6^ cells/ml. In addition, non-essential amino acids (Gibco, 11146-050), Penicillin–Streptomycin (Gibco, 15140163), β-mercaptoethanol (Gibco, 21985-023) and sodium pyruvate (Gibco, 11360-070) were also added in order to maintain cell growth. Tag-it Violet™ - labeled T cells were activated with coated purified anti-mouse CD3 (Tonbo Biosciences, 70-0031) and purified anti-mouse CD28 (Tonbo Biosciences, 70-0281) in U-bottom 96-well plates and co-cultured with sorted MDSCs from old or young mice at the ratio of 1:1 (50 × 10^3 ^T cells : 50 × 10^3^ MDSCs). After 4 days, cells were harvested and blocked with anti-mouse CD16/32 (BioLegend, 101302), then the Fixable Viability Dye eFluor™ 450 (eBioscience, 65-0863) in combination with fluorescently labeled antibodies against CD4 (BioLegend, 100512) and CD8 (BioLegend, 100709) were used for staining purposes, and processed with BD FACSAria™ III and analyzed by FlowJo software version 10.0.7 (Tree Star Inc.). The aged mice used for T cell suppression assay were 20 months old, while the young mice used as controls were 8 weeks old.

### Determination of ROS generation

Intracellular ROS were detected by probe 2’,7’-dichlorofluorescein diacetate (DCFH-DA, Sigma-Aldrich, D6883). In brief, selected cells were incubated with 10 μM DCFH-DA for 30 min at 37 °C. Fluorescence minus one (FMO)— DCFH controls were used to establish photomultiplier tube voltages. Fluorescence intensity (Ex/Em = 488/525 nm) was detected with BD FACSAria™ III and analyzed by FlowJo software version 10.0.7 (Tree Star Inc.). The aged mice used for the determination of ROS generation were 20 months old, while the young mice used as controls were 8 weeks old.

### RNA velocity analysis

RNA velocity has opened up new ways of studying cellular differentiation in single-cell RNA-sequencing data. In the present study, we used scVelo package (version 0.2.4) to analyze MDSC generation. The data were processed using default parameters following preprocessing as described in scVelo package. Analysis of cellular trajectory inference by RNA velocity was performed using dynamical model, which is a generalization of the original RNA velocity method and allows for characterization of multiple transcriptional states^55^. In addition, latent time analysis, pseudotime analysis and velocity confidence were computed using default parameters.

### Pseudotime trajectory analysis

The R package Monocle 2 (2.22.0) was used to reconstruct the differentiation trajectories of both PMN-MDSCs and M-MDSCs, respectively^56^. Briefly, the generated Seurat object was transformed into Monocle object with the “GetAssayData” and “newCellDataSet” functions. Next, we used Monocle’s “orderCells” function to arrange cells along the pseudotime axis which revealed the differential trajectory. After pseudotime was determined, the pseudotime kinetics of different marker genes were displayed using the “plot_genes_in_pseudotime” function in the R package. In addition, “plot_pseudotime_heatmap” function in the R package was used to generate the pesudotime heat map of the top 50 genes with the most significant alterations. Trajectory analysis and pseudotime diagrams of human myeloid cells were generated using Monocle 3 (1.3.7).

### MDSC signature gene scoring of human bone marrow samples

Single-cell transcriptomic data of aged human myeloid cells were downloaded with associated metadata from the GSE253355 dataset in the Gene Expression Omnibus (GEO)^57^. Additionally, single-cell transcriptomic data of human neutrophils and monocytes across various ages, sourced from Jong et al.^58^, Oetjen et al.^59^ and Wu et al.^60^ were downloaded with associated metadata from Deeply Integrated human Single-Cell Omics database (DISCO) (https://www.immunesinglecell.org/). The Single-cell transcriptomic data were processed in R version 4.4.0. Seurat’s “AddModuleScore” function was used to score all of the cells in the analysis using the established MDSC-specific signature genes in the current study (Supplementary Data 4).

### Statistics

All data were presented as mean ± SD. Significance was assessed using two-tailed Student’s *t* test for comparison between two groups or one-way analysis of variance (ANOVA) for comparison between more than two groups. A confidence level above 95% (*P* < 0.05) was determined to be significant.

## Supplementary information

Supplementary Figures & Supplementary Data Legend

Supplementary Data 1

Supplementary Data 2

Supplementary Data 3

Supplementary Data 4

Supplementary Data 5

Supplementary Data 6

## Data Availability

Sequencing datasets have been deposited in the GEO database under the accession number GSE275786.
