# Supplementary material for: Defining the marker and developmental trajectory of myeloid-derived suppressor cells in aging by single-cell transcriptomics
Source: NPJ Aging. 2025 Dec 24;12(1):18. doi: 10.1038/s41514-025-00317-x (PMC12855942; doi:10.1038/s41514-025-00317-x)
Supplement: Supplementary file 1 — Supplementary Figures & Supplementary Data Legend [file 41514_2025_317_MOESM1_ESM.pdf]

Defining the marker and developmental trajectory of myeloid-derived suppressor cells in aging by single-cell transcriptomics

Yaru Su[2*], Ruimin Wu[3*], Haochen Ai[3*], Zhaoming Zhong[1], Lin Zou[4], Zihan Wang[1], Kewu Tu[1], Lingzheng Tang[1], Jiawen Gao[1], Yusheng Huang[1], Congrui Liao[1], Guanhai Zeng[1], Hongyang Zhang[2#], Jian Jin[1#], and Siyuan Zhu[1#]

1. Division of Spine Surgery, Department of Orthopedics, Nanfang Hospital, Southern Medical University, Guangzhou, China.
2. Department of Ophthalmology, Nanfang Hospital, Southern Medical University, Guangzhou, China.
3. The First School of Clinical Medicine, Southern Medical University, Guangzhou, China.
4. Department of Orthopedics, Taihe People's Hospital, Guangzhou, China.

# Corresponding authors:

1. Siyuan Zhu,
Division of Spine Surgery, Department of Orthopedics, Nanfang Hospital, Southern Medical University, 1838 North Guangzhou Avenue, Guangzhou 510515, P. R. China
E-mail address: zsyneo@163.com
2. Jian Jin,
Division of Spine Surgery, Department of Orthopedics, Nanfang Hospital, Southern Medical University, 1838 North Guangzhou Avenue, Guangzhou 510515, P. R. China
E-mail address: 38323580@qq.com
3. Hongyang Zhang,
Department of Ophthalmology, Nanfang Hospital, Southern Medical University, 1838 North Guangzhou Avenue, Guangzhou 510515, P. R. China
E-mail address: hy3005716@163.com

*** Yaru Su, Ruimin Wu and Haochen Ai contributed equally to this work.**

Supplementary Figures

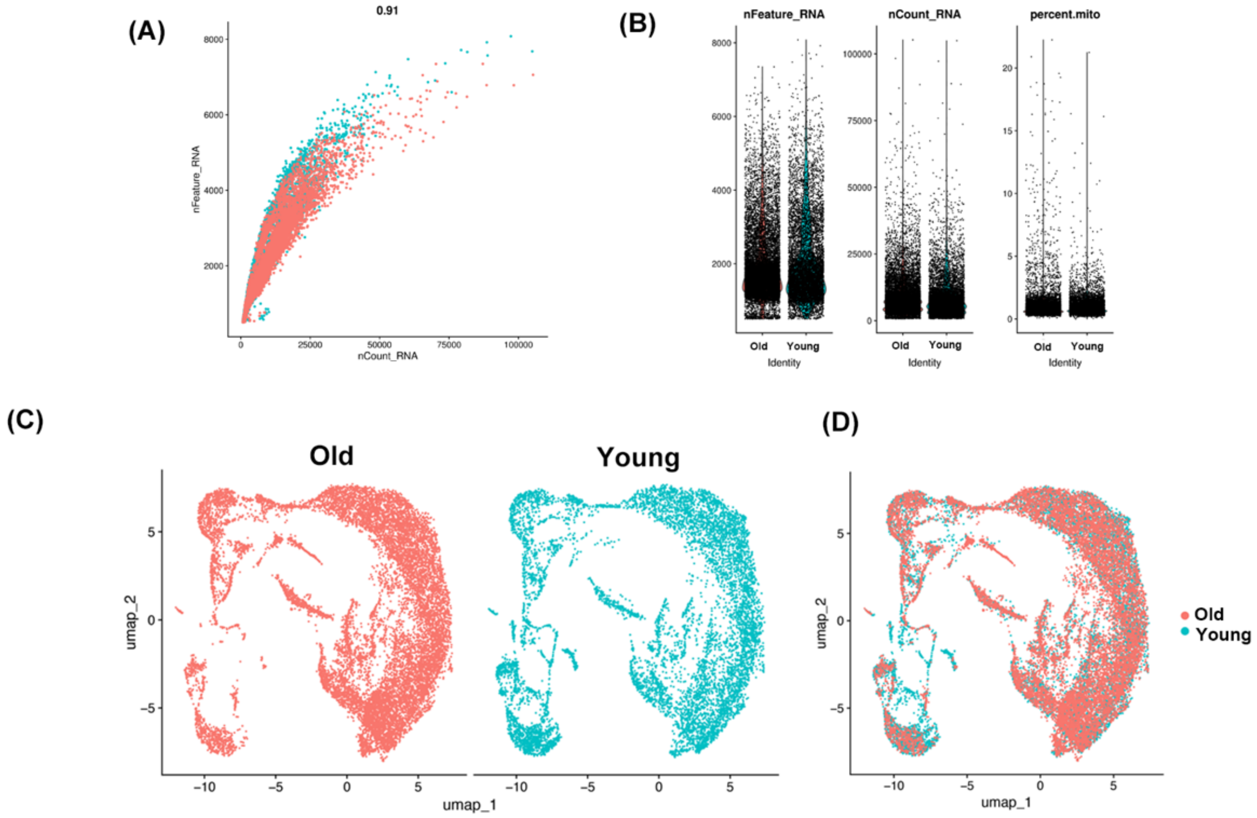

Figure S1. **(A)** The basic information of the filtered cells in each sample was shown in scatter plots. **(B)** Violin plots of the number of unique genes detected in each cell (nFeature_RNA), the total number of molecules detected within a cell (nCount_RNA), and the percentage of reads that map to the mitochondrial genome (percent.mto) after filtering. **(C-D)** Distribution of cells from different samples, which were shown using UMAP plots.

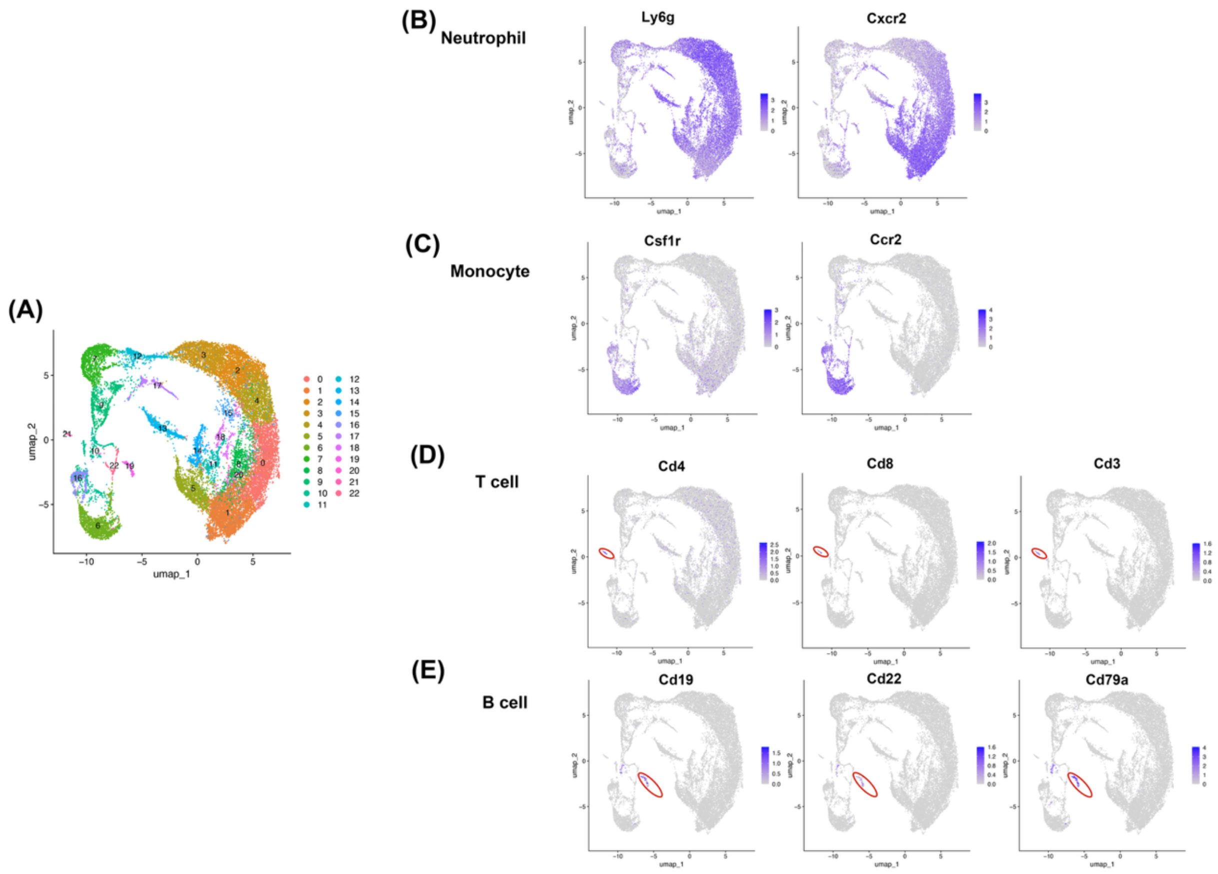

Figure S2. (A) UMAP plot result showed 23 distinct subclusters. **(B)** Ly6g, Cxcr2 were used to identify neutrophils. **(C)** Csf1r, Ccr2 were used to identify monocytes. **(D)** Cd4, Cd8, and Cd3 were used to identify T cells. **(E)** Cd19, Cd22, and Cd79a were used to identify B cells.

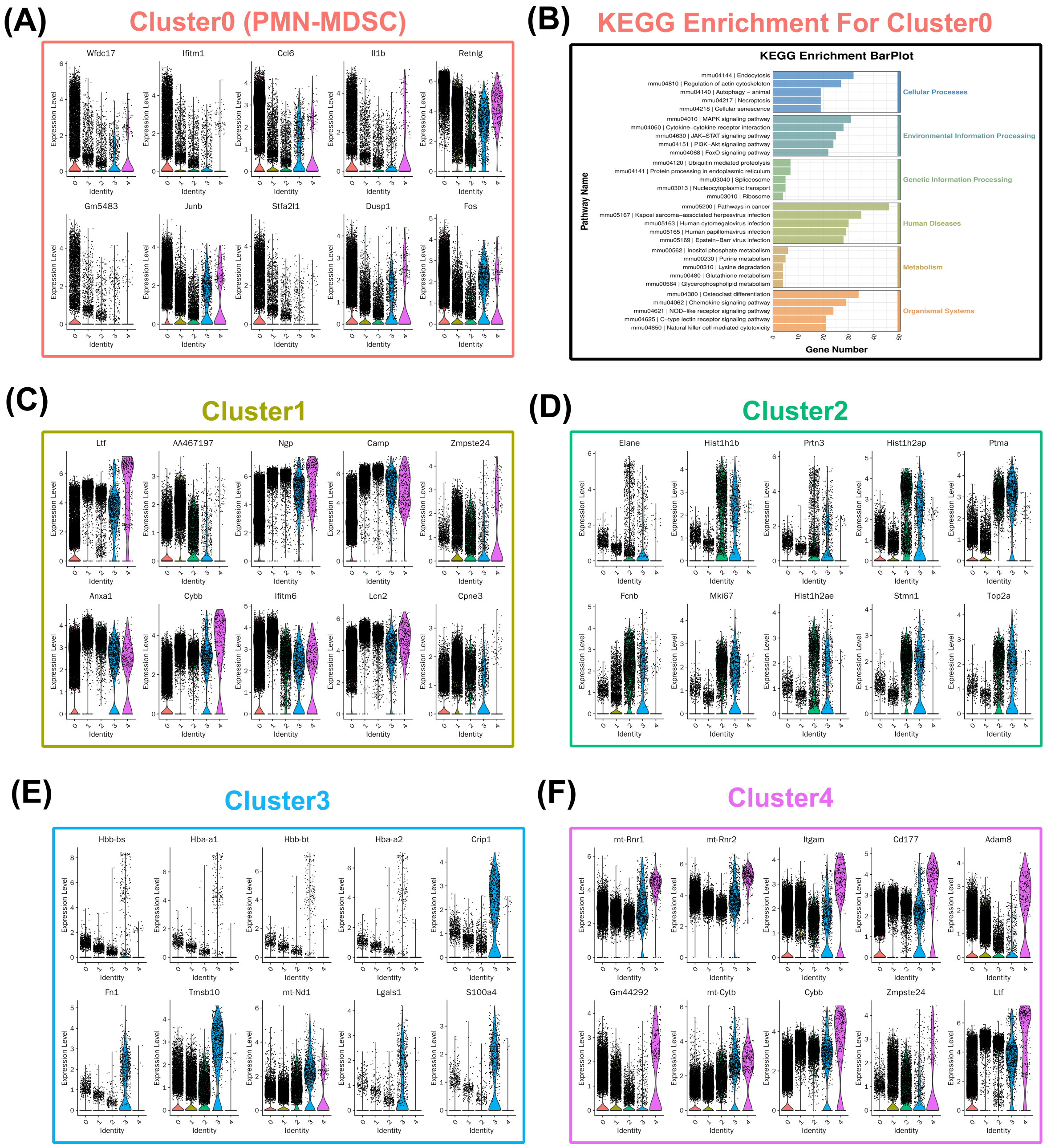

Figure S3. (A) Violin plots showed top 10 marker genes of Cluster 0 (PMN-MDSC). **(B)** KEGG enrichment analysis of the marker genes of Cluster 0. **(C-F)** Violin plots showed top 10 marker genes of Cluster 1, Cluster 2, Cluster 3, and Cluster 4.

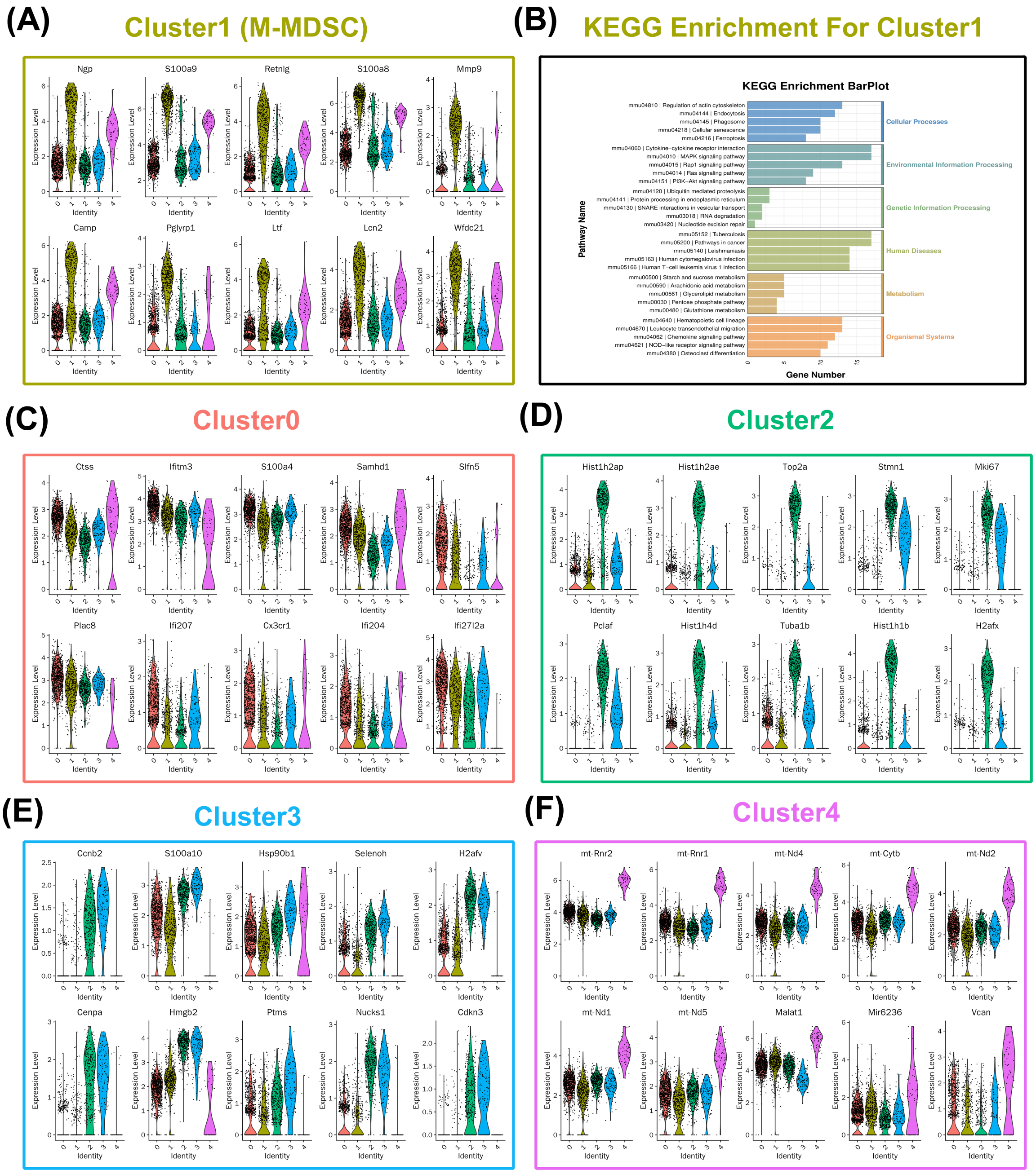

Figure S4. (A) Violin plots showed top 10 marker genes of Cluster 1 (M-MDSC). **(B)** KEGG enrichment analysis of the marker genes of Cluster 1. **(C-F)** Violin plots showed top 10 marker genes of Cluster 0, Cluster 2, Cluster 3, and Cluster 4.

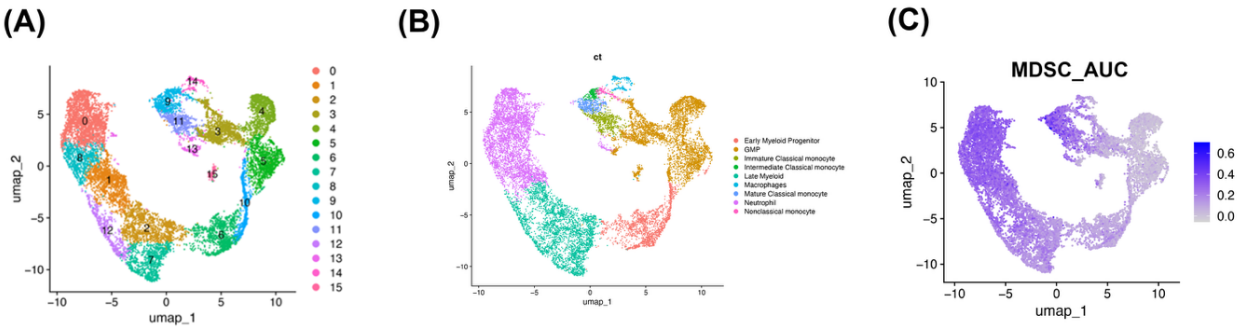

Figure S5. scRNAseq datasets of myeloid cells derived from aged males and females. (A-B) Nine myeloid cell subclusters were identified, such as early myeloid progenitors, granulocyte-monocyte progenitors, immature classical monocytes, late myeloid, intermediate monocytes, macrophages, mature classical monocytes, neutrophils, nonclassical monocytes. **(C)** The expressions of MDSC signature genes in neutrophils and monocytes displayed a dispersed or non-specific pattern.

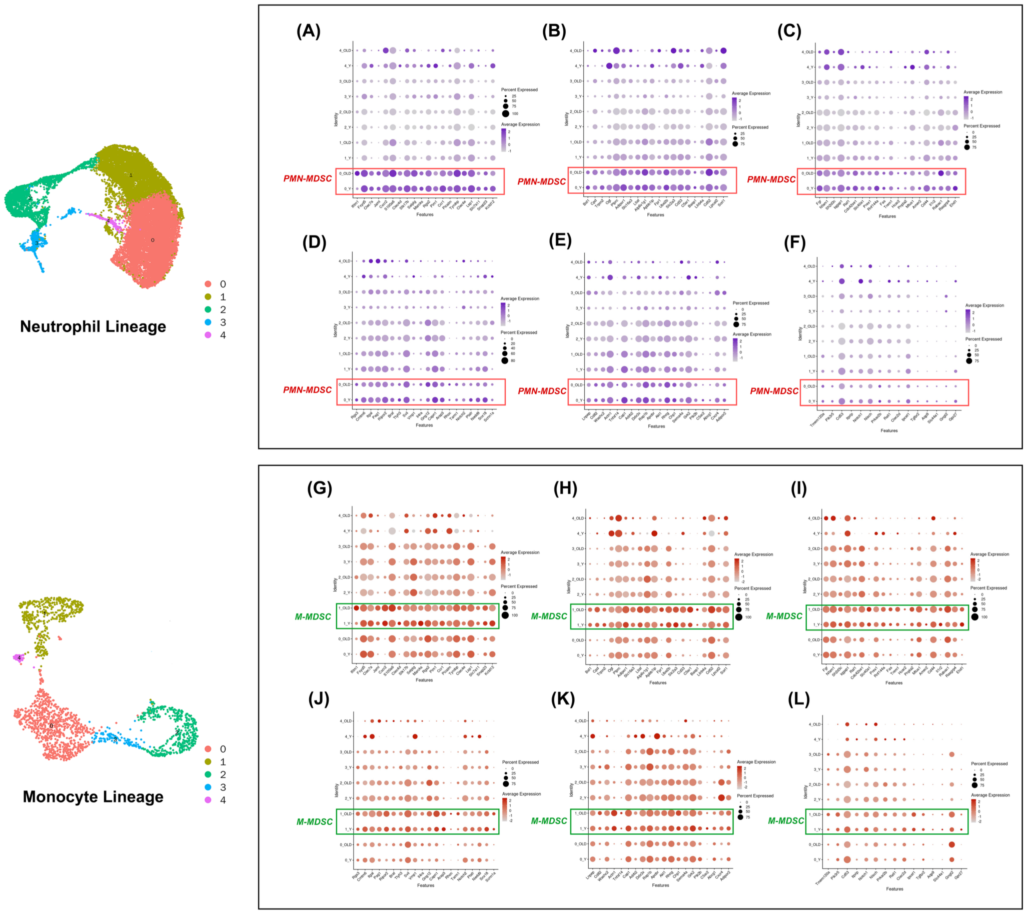

Figure S6. (A-F) Dot plots demonstrated the expressions of surface markers in different neutrophilic cell subclusters in aged or young mice. **(G-L)** Dot plots demonstrated the expressions of surface markers in different monocytic cell subclusters in aged or young mice.

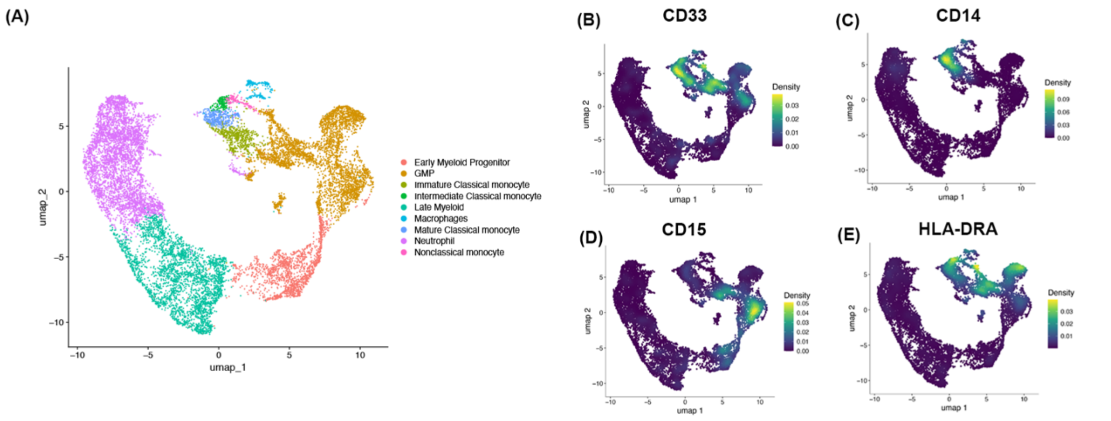

Figure S7. E-MDSCs may not exist in the healthy elderly population. (A) Single-cell transcriptomic data of healthy aged human myeloid cells revealed nine distinct myeloid cell clusters. **(B-E)** Gene expression density plot showed the expression of Cd33, Cd14, Cd15 and HLA-DRA, according to the phenotype of E-MDSCs (CD3$^-$CD14$^-$CD15$^-$CD19$^-$CD56$^-$HLA-DR$^-$CD33$^+$). However, no subpopulation simultaneously exhibits the CD3$^-$CD14$^-$CD15$^-$CD19$^-$CD56$^-$HLA-DR$^-$CD33$^+$ signature.

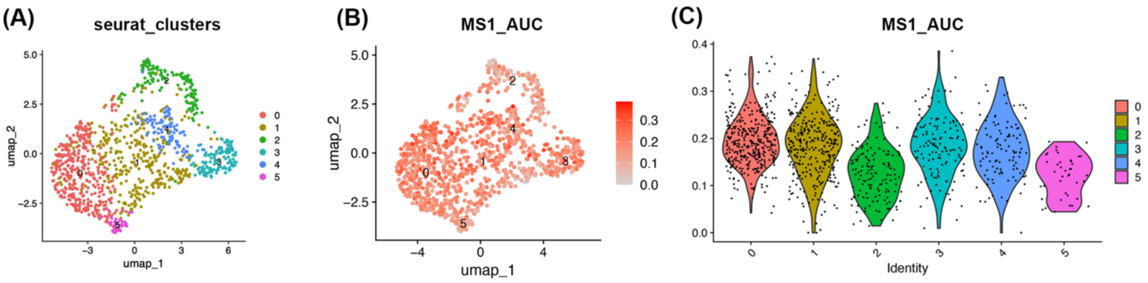

Figure S8. MS1 cells may not exist in the healthy elderly population. (A-C) According to 15 MS1 marker genes reported by Reyes et al., we applied “AUCell” to score the characteristic gene set of MS1 cells, but found that these methods could not identify MS1 cells in healthy human monocytic lineages. Therefore, we consider that MS1 cells may not exist in the healthy elderly population.

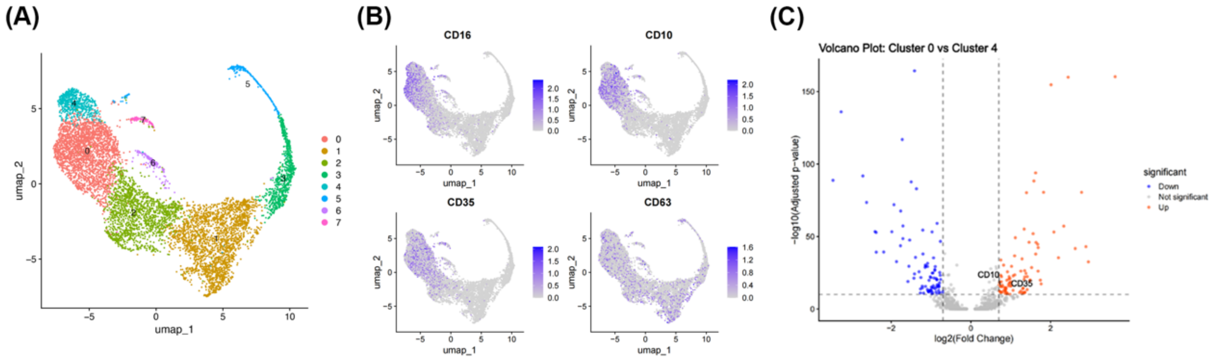

Figure S9 In healthy individuals, LDNs are likely intermingled with mature neutrophils and lack a distinct functional role. (A-B) The marker genes of LDNs were visualized in the aged human neutrophil dataset and found their expression in both cluster 0 and cluster 4, with more prominent expression in cluster 0. **(C)** Given that we have previously identified cluster 4 as PMN-MDSCs, we performed differential gene expression analysis (volcano plot) between cluster 0 and cluster 4. The reported LDN markers CD10 and CD35 both showed significant differences. This suggests that in healthy elderly individuals, PMN-MDSCs and LDNs may not represent the same cell population.

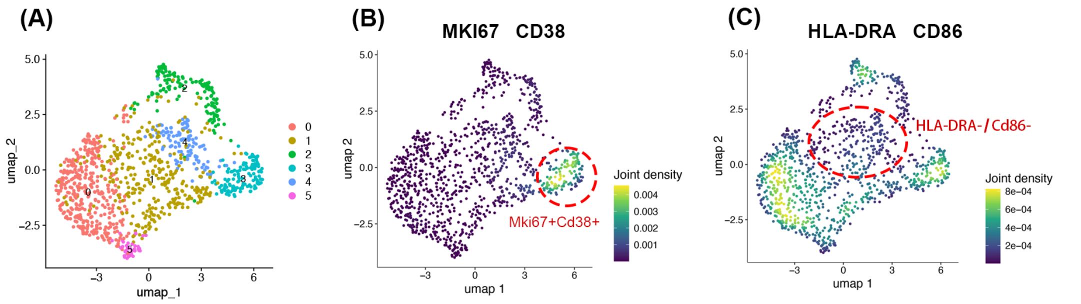

Figure S10 Exhausted monocytes might not be present in the healthy elderly population. (A-C) Key features of monocyte exhaustion including reduced differentiation, pathogenic inflammation and immune suppression which is similar with M-MDSCs. Exhausted monocytes are characterized by the molecular signature MKI67^{hi} CD38^{hi} CD86^{low}HLA-DRA^{low}, which corresponds to their less mature, pathogenic inflammatory, and immunosuppressive properties, respectively. So we tried to identify exhausted monocytes in aged human monocyte dataset, however, no monocyte subpopulation simultaneously exhibits the MKI67^{hi} CD38^{hi} CD86^{low}HLA-DRA^{low} signature.

Supplementary Data Legend

Supplementary Data 1. The marker gene list of 22 subclusters.

Supplementary Data 2. The marker gene list of PMN-MDSC in aging process.

Supplementary Data 3. The marker gene list of M-MDSC in aging process.

Supplementary Data 4. The signature gene list of MDSC in aging process.

Supplementary Data 5. MDSC signature genes which encode the membrane-expressed proteins.

Supplementary Data 6. The primer pair sequences.
